# The Effect of Anti‐Activin Receptor Type IIA and Type IIB Antibody on Muscle, Bone and Blood in Healthy and Osteosarcopenic Mice

**DOI:** 10.1002/jcsm.13718

**Published:** 2025-01-30

**Authors:** Frederik Duch Bromer, Andreas Lodberg, Marco Eijken, Christian Brix Folsted Andersen, Mathias Flensted Poulsen, Jesper Skovhus Thomsen, Annemarie Brüel

**Affiliations:** ^1^ Department of Biomedicine Aarhus University Aarhus Denmark; ^2^ Department of Clinical Medicine Aarhus University Aarhus Denmark; ^3^ Department of Endocrinology and Internal Medicine Aarhus University Hospital Aarhus Denmark

**Keywords:** haematopoiesis, activin, bimagrumab, disuse, osteoporosis, sarcopenia, Smad2

## Abstract

**Background:**

Anti‐Activin Receptor Type IIA and Type IIB antibody (αActRIIA/IIB ab) is a recently developed drug class that targets the activin receptor signalling pathway. Inhibition of receptor ligands (activins, myostatin, growth differentiation factor 11, etc.) can lead to skeletal muscle hypertrophy, bone formation, and increased haematopoiesis. Despite the αActRIIA/IIB ab, bimagrumab, having progressed to clinical trials, two crucial questions about αActRIIA/IIB ab therapy remain: Does αActRIIA/IIB ab influence bone metabolism and bone strength similarly to its generic classmates (activin receptor‐based ligand traps)? Does αActRIIA/IIB ab affect red blood cell parameters, thereby increasing the risk of thromboembolism, similar to its generic classmates? Therefore, the aim of the present study was to investigate the therapeutic potential of αActRIIA/IIB ab in a mouse model of concurrent sarcopenia and osteopenia and to investigate the effect on bone and haematopoiesis in more detail.

**Methods:**

In C57BL/6JRj mice, combined sarcopenia and osteopenia were induced locally by injecting botulinum toxin A into the right hindlimb, resulting in acute muscle paresis. Immediately after immobilization, mice received twice‐weekly intraperitoneal injections with αActRIIA/IIB ab (10 mg/kg) for 21 days, after which they were sacrificed. Muscle mass, skeletal muscle fibre size and Smad2 expression were analysed in the rectus femoris and gastrocnemius muscles. Bone mass and bone microstructure were analysed in the trabecular bone at the distal femoral metaphysis, while the cortical bone was analysed at the femoral mid‐diaphysis. In a substudy, the effect on haematopoiesis was explored 2 and 7 days after a single αActRIIA/IIB ab (30 mg/kg) injection in C57BL/6JRj mice.

**Results:**

αActRIIA/IIB ab caused a large increase in muscle mass in both healthy (+21%) and immobilized (sarcopenic and osteopenic) (+12%) mice. Furthermore, αActRIIA/IIB ab increased trabecular bone (bone volume fraction) for both healthy (+65%) and immobilized (+44%) mice. For cortical bone, αActRIIA/IIB ab caused a small, but significant, increase in bone area (+6%) for immobilized mice, but not for healthy mice. Treatment with αActRIIA/IIB ab did not change red blood cell count, haemoglobin concentration or mean cell volume after either 2 or 7 days.

**Conclusions:**

Treatment with αActRIIA/IIB ab caused a significant increase in both skeletal muscle mass and bone parameters in both healthy and immobilized mice, suggesting a potential in the treatment of concurrent osteopenia and sarcopenia. Interestingly, the bone anabolic effect of the treatment was much more pronounced on trabecular bone than on cortical bone. There was no pronounced effect of short‐term treatment on haematopoiesis.

## 1 Introduction

Muscle wasting (sarcopenia) and osteoporosis are both conditions frequently found in the elderly population—and as life expectancy is increasing, both conditions become more common [1]. However, concurrent atrophy of skeletal muscle and bone is not only caused by advanced age but can also be due to physical inactivity or disuse [2].

Osteoporosis is a debilitating skeletal condition characterized by low bone mineral density (BMD) and microstructural bone deterioration leading to increased bone fracture risk [3]. Whereas osteoporosis comprises a deterioration of the skeleton, sarcopenia is a condition defined by loss of skeletal muscle mass, strength and function [4], which inflicts severe restrictions on quality of life [5].

For years, research on the interaction between muscle and bone has focused on the mechanical coupling between the tissues [6], but the discovery of myokines indicates that there is more to bone–muscle interaction than simply mechanical forces [7]. Current pharmaceuticals targeting bone loss have limited effect on skeletal muscle [8], and there are no approved pharmaceutical options for sarcopenia [9]. Therefore, therapies that can induce skeletal muscle hypertrophy and increase bone formation simultaneously are needed.

In recent years, inhibitors of the activin receptor signalling pathway (IASPs) have attracted attention due to their potent anabolic effect on muscle, bone and haematopoiesis [10].

IASPs inhibit the activin receptor signalling pathway either by targeting activin receptor ligands or by blocking the activin receptor complex directly [10]. The activin receptor complex is a transmembrane heteromer comprised of two Type I and two Type II receptors, each with several different subtypes [11]. As ligand affinity is highest for Type II receptors, activation of the pathway usually starts with a ligand (e.g., myostatin, activin or growth differentiation factor 11 (GDF‐11)) binding to a Type II receptor, which then recruits and activates a Type I receptor [11], resulting in downstream activation of Smad2/Smad3 transcription factors [12]. Inhibition of this pathway ultimately leads to muscle hypertrophy, increased bone formation and, in some cases, increased haematopoiesis [13, 14, 15].

The Anti‐Activin Receptor Type IIA and Type IIB antibody (αActRIIA/IIB ab), bimagrumab, is a human antibody that binds and blocks the ligand‐binding site on both activin receptor Type IIA and Type IIB, thereby inhibiting signal propagation downstream of the receptors [16, 17]. Multiple preclinical and clinical studies have shown that treatment with bimagrumab causes hypertrophy of skeletal muscle and increases lean body mass [16, 17, 18, 19, 20, 21]. However, the evidence for an improvement in muscle function is less clear, with clinical studies reporting conflicting effects on 6‐min walking capability [18, 22]. Currently, a clinical trial is investigating whether bimagrumab can preserve muscle mass during pharmaceutically induced weight loss (NCT05616013).

Although the hypertrophic effect of αActRIIA/IIB ab on muscle tissue is well documented, few studies have investigated its effect on bone and blood. αActRIIA/IIB ab could potentially be used to simultaneously treat the sarcopenia and osteoporosis that occur with advanced age or physical inactivity. Furthermore, since other IASPs stimulate haematopoiesis to a degree where the risk of thrombosis must be considered, it is also imperative to investigate in more detail the effects of αActRIIA/IIB ab on haematopoiesis [14].

The aim of the present study was to explore the effect of αActRIIA/IIB ab treatment on muscle and bone tissue in both healthy mice and mice suffering from immobilization‐induced concurrent sarcopenia and osteopenia. Furthermore, we investigated whether short‐term treatment with αActRIIA/IIB ab affected haematopoiesis in mice.

## 2 Materials and Methods

### 2.1 Preparation of Anti‐Activin Receptor Type IIA/IIB Antibody

Constructs containing αActRIIA/IIB ab light chain and IgG1 heavy chain were transfected into Chinese hamster ovary (CHO) cells, and αActRIIA/IIB ab was purified from the conditioned medium using affinity chromatography and exchanged into phosphate‐buffered saline (PBS). A detailed description of the production and purification of αActRIIA/IIB ab can be found in the Supporting Information section.

### 2.2 Animals and Procedures for Immobilization Study

Sixteen‐week‐old female C57BL/6JRj mice (Janvier Labs, Le Genest‐Saint‐Isle, France) were stratified by body weight (BW) into five groups: baseline (*n* = 12), ambulating + vehicle (Amb) (*n* = 12), ambulating + αActRIIA/IIB ab (Amb‐αActRIIA/IIB ab) (*n* = 12), botulinum toxin A (BTX)‐immobilized + vehicle (BTX) (*n* = 12) and BTX‐immobilized + αActRIIA/IIB ab (BTX‐αActRIIA/IIB ab) (*n* = 12). The animals were housed with a 12‐h day/night cycle in individually ventilated cages at 22°C, a humidity of 50% and ad libitum access to standard chow and water.

On Day 0, animals were anaesthetized with 3% isoflurane (IsoFlo Vet; Orion Pharma Animal Health, Copenhagen, Denmark), and their right hindlimb was shaved. Sarcopenia and osteopenia were induced by injection of BTX [23], which is an established model of immobilization‐induced bone loss [24]. BTX (2 U/100 g BW in total; Allergan, Irvine, CA) was injected into both the quadriceps (10 μL) and gastrocnemius (10 μL) muscles of the right hindlimb. Ambulating animals were injected with an equivalent volume of isotonic NaCl. The effect of the BTX injections was evaluated using the gait ability score developed by Warner et al. [25]. In brief, each mouse was given a score from 2 points (*full movement*) to 0 points (*no movement*) on 5 different mobility aspects for a total possible score ranging from 0 (*complete paralysis*) to 10 (*normal use*).

Animals were weighed weekly. Starting from the day of BTX injection, animals were injected intraperitoneally (ip) twice weekly with either αActRIIA/IIB ab (10 mg/kg) or an equal volume vehicle (PBS).

For dynamic histomorphometry, animals were labelled subcutaneously (sc) with tetracycline (20 mg/kg, T3383; Sigma‐Aldrich, St. Louis, Missouri, United States) on Day −4 and with alizarine red (20 mg/kg, A3882; Sigma‐Aldrich, St. Louis, Missouri, United States) 8 and 4 days prior to euthanization.

After 21 days, the animals were anaesthetized by inhalation of 3% isoflurane and sacrificed by removal of the heart. The right rectus femoris muscle was isolated, weighed and fixed in 4% formaldehyde for 48 h before being transferred to 70% ethanol. Subsequently, the right gastrocnemius muscle was isolated, weighed and snap‐frozen in liquid nitrogen before being stored at −80°C. The right femur was removed, length measured by a digital calliper and stored in Ringer's solution at −20°C. The tibia was removed and fixed in 4% formaldehyde for 48 h before being transferred to 70% ethanol.

One animal from the BTX‐αActRIIA/IIB ab group was excluded and euthanized prior to the study end due to malocclusion of the teeth, considered unrelated to the treatment. The protocol was approved by the Danish Animal Experiments Inspectorate (Permit: 2023‐15‐0201‐01404).

### 2.3 Western Blot Analysis

The gastrocnemius muscles were homogenized in a lysis buffer, agitated and centrifuged, and the supernatant was collected. Thirty micrograms of protein from each animal was loaded onto a gel for SDS‐PAGE separation and stain‐free visualization on an imaging system (Gel Doc EZ Imager; Bio‐Rad Laboratories, Hercules, California, United States). Samples were incubated with primary antibody against Smad2 or phosphorylated (p)Smad2 and secondary antibody against rabbit IgG. Bands were quantified using FIJI and normalized to the total protein load, as determined from the stain‐free gel analysis [26]. A detailed description of immunoblotting procedures and materials can be found in the Supporting Information section.

### 2.4 Smad2 Protein Expression After Short‐Term Immobilization

To further investigate the short‐term effect of immobilization, a substudy was conducted in which twenty‐four 16‐week‐old female C57BL/6JRj mice (Janvier Labs, Le Genest‐Saint‐Isle, France) were randomized into the same groups as in the original immobilization study: Amb (*n* = 6), Amb‐αActRIIA/IIB ab (*n* = 6), BTX (*n* = 6) and BTX‐αActRIIA/IIB ab (*n* = 6). The animals treated with αActRIIA/IIB ab received a single ip injection of 30 mg/kg on Day 0, and all animals were euthanized after 3 days. The gastrocnemius muscle was removed and snap‐frozen in liquid nitrogen.

### 2.5 Muscle Scan and Histology

The rectus femoris muscle was cut cross‐sectionally at the midpoint and scanned in a flatbed scanner (Perfection 3200 Photo; Seiko Epson, Nagano, Japan), and gross muscle cross‐sectional area (CSA) was determined. Subsequently, the rectus femoris muscle was embedded in Technovit 7100 (Axlab, Vedbæk, Denmark), cut into 4‐μm‐thick sections on a microtome (Jung RM2065; Leica Instruments, Nussloch, Germany), mounted on glass slides and stained with Masson's trichrome for stereological analysis. Muscle fibre CSA was estimated using the 2D‐Nucleator principle [27]. For each animal, an average of 182 counts were performed.

### 2.6 Dual‐Energy X‐Ray Absorptiometry (DEXA)

DEXA of the femur was obtained (Sabre XL; Norland Stratec, Birkenfeld, Germany) at a scan speed of 3 mm/s and a spatial resolution of 0.1 mm × 0.1 mm. Bone mineral content (BMC) and areal bone mineral density (aBMD) were determined by the software supplied with the scanner.

### 2.7 Mechanical Testing

Bone strength was determined at the femoral mid‐diaphysis and the femoral neck using a materials testing machine, as previously described [28]. In brief, downward pressure was applied to the midpoint of the femur until fracture in a 3‐point bending test. Subsequently, load was applied to the femoral head until the fracture of the femoral neck.

### 2.8 Microcomputed Tomography (μCT)of Bone

μCT (μCT35; Scanco Medical AG, Brüttisellen, Switzerland) was performed using an x‐ray tube voltage of 55 kVp. The distal femoral metaphysis and femoral mid‐diaphysis were imaged using resolutions of 3.5 and 7 μm, respectively, and an integration time of 800 and 300 ms, respectively. A detailed description of μCT can be found in the Supporting Information section.

### 2.9 Bone Preparation, Cell Counting and Dynamic Histomorphometry

For microscopy, 200‐μm‐thick femoral mid‐diaphyseal cross‐sections and 7‐μm‐thick femoral and tibial longitudinal sections were prepared. The femoral samples were left unstained, while the tibial samples were stained with either Masson–Goldner trichrome or enzymatically stained for tartrate‐resistant acid phosphatase (TRAP).

At the proximal tibial metaphysis, osteoid‐covered bone surfaces (OS/BS), osteoblast‐covered bone surfaces (Ob.S/BS) and medullary adipocyte density were estimated on Masson–Goldner trichrome–stained sections, while osteoclast‐covered bone surfaces (Oc.S/BS) were estimated on sections stained for TRAP. Mineralizing surface (MS/BS), mineral apposition rate (MAR) and bone formation rate (BFR/BS) were estimated at the femoral mid‐diaphysis and metaphysis [29]. A detailed description of preparation for histological analyses can be found in the Supporting Information section.

### 2.10 Haematopoiesis in Mice

Since multiple types of IASPs have been shown to affect haematopoiesis, two substudies were performed in 7‐week‐old and 19‐week‐old mice to investigate the effect of short‐term αActRIIA/IIB ab treatment on haematopoiesis independent of immobilization. Mice of different ages were used as haematological parameters may vary with age [30].

#### 2.10.1 Haematological Parameters in 7‐Week‐Old Mice

Seven‐week‐old acclimatized C57BL/6JRj mice (Janvier Labs, Le Genest‐Saint‐Isle, France) were stratified by BW into four groups (*n* = 6 for each group) and injected ip with either αActRIIA/IIB ab (30 mg/kg) or equal volume vehicle (PBS) for controls (Ctrls). Two groups were euthanized 2 days after αActRIIA/IIB ab treatment (Group 1: 2d‐Ctrl and Group 2: 2d‐αActRIIA/IIB ab) and two groups 7 days after treatment (Group 3: 7d‐Ctrl and Group 4: 7d‐αActRIIA/IIB ab).

Housing, food conditions and euthanasia were as described above. Whole blood was collected with 1.5 g/L K‐EDTA and placed on a rocking mixer for approximately 3 h before it was analysed in doublets by a complete blood count test (ProCyte DX; Idexx, Maine, United States). After collecting blood, the left rectus femoris muscle was isolated and weighed. Then the left hindlimb was detached, the skin was removed and μCT was performed as described below. Finally, the spleen was removed and weighed.

To confirm the effect of treatment, μCT (vivaCT 80; Scanco Medical AG, Brüttisellen, Switzerland) of the muscles in the left hindlimb was performed using an x‐ray tube voltage of 45 kV and a current of 177 μA, a voxel size of 62.4 μm, an integration time of 200 ms and 500 projections per scan. A 5‐mm‐high volume of interest (VOI) was placed distally from the most proximal part of the fibula, and calf muscle volume was ascertained.

#### 2.10.2 Haematological Parameters in 19‐Week‐Old Mice

Two age‐matched groups (Ctrl: *n* = 5 and αActRIIA/IIB ab: *n* = 5) of 19‐week‐old female C57BL/6JRj mice (Janvier Labs, Le Genest‐Saint‐Isle, France) were studied. Housing, treatment and collection of whole blood were performed as described above. Mice were euthanized 2 days after treatment.

### 2.11 Statistical Analysis

A priori sample size calculations (power = 0.8) showed that a 12% difference in mid‐femoral mechanical strength could be detected with *n* = 12 mice. Furthermore, a difference of 10% in red blood cell count (RBC) could be detected with *n* = 5 mice.

Statistical analysis was performed using GraphPad Prism 10.0.1. The normality of data was assessed by a QQ‐plot of the residuals. In the immobilization study, for normally distributed data, parametric two‐way analysis of variance (ANOVA) was performed, followed by a Holm–Sidak analysis for multiple comparisons to investigate the effect between Amb versus BTX, Amb versus Amb‐αActRIIA/IIB ab and BTX versus BTX‐αActRIIA/IIB ab. If the data was not normally distributed, the Kruskal–Wallis test was performed, followed by Dunn's test. For the haematological substudies, an unpaired *t*‐test was used for normally distributed data, and a Mann–Whitney test was used if the data was not normally distributed. The baseline was not included in the statistical analysis but is shown in tables and figures. The significance level was set at 5%. Data is presented as relative values in the text and as mean ± SD in tables and figures.

## 3 Results

### 3.1 Activin Type II Receptor Neutralization Assay

**FIGURE 1:**
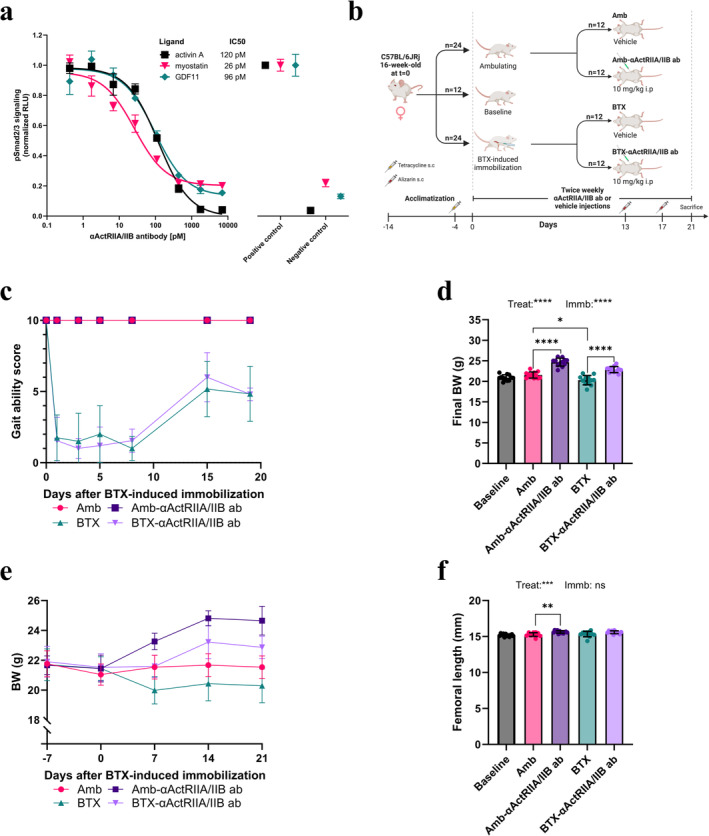
Study setup, gait, bodyweight and bone length. (a) αActRIIA/IIB ab inhibition of the (CAGA)12‐luciferase signalling in Smad2/3 sensitive reporter cells induced by activin A (1 ng/mL), myostatin (1 ng/mL) and GDF 11 (1 ng/mL). Smad2/3 signal strength was normalized to uninhibited signalling strength (normalized relative light units, RLU). Each analysis was run in triplicates. Positive control shows maximum signal strength without αActRIIA/IIB ab inhibition. Negative control shows a background signal with no stimulation by activin A, myostatin or GDF‐11. (b) Study set‐up for the 21‐day immobilization study. Created with BioRender. (c) Gait ability score. Animals received BTX at *t* = 0. (d) Final body weight (BW). (e) BW over time. (f) Final femoral length. Data is presented as mean ± SD. Key: Treat: effect of αActRIIA/IIB ab treatment in two‐way ANOVA. Immb: effect of BTX immobilization in two‐way ANOVA. * *p* < 0.05, **p < 0.01, ****p < 0.0001, ns = not significant.

The bioactivity of αActRIIA/IIB ab was verified using a (CAGA)12‐luciferase‐based bioassay of pSmad2/3 signalling. αActRIIA/IIB ab inhibited activin A (1 ng/mL), myostatin (1 ng/mL) and GDF11 (1 ng/mL) induced pSmad2/3 signalling with a half‐maximal inhibitory concentration (IC50) of 120, 26 and 96 pM, respectively (Figure 1a).

### 3.2 Gait Ability Score, BW and Femoral Length

A visual representation of the experimental groups and study setup in the 21‐day immobilization study can be seen in Figure 1b. Hindlimb injection with BTX is an established model of immobilization in animal studies [23, 24]. The neurotoxin BTX induces paresis by blocking the release of acetylcholine from the neuromuscular junction, thereby inhibiting the activation of the skeletal muscle cells [31]. This paresis results in a substantial loss of both muscle and bone mass in the affected limb [25, 28].

The effect of BTX was confirmed when injected animals had a reduction in gait ability after 1 day with a minimum score around Day 7 (Figure 1c). After 7 days, the gait ability score increased but did not reach the level of ambulating animals. Treatment with αActRIIA/IIB ab did not affect gait ability.

At the end of the study, BTX animals had a significantly lower final BW (−6%) than their ambulating counterparts (Figure 1d,e). αActRIIA/IIB ab treatment increased final BW significantly in both ambulating (+14%) and immobilized (+13%) mice.

BTX did not affect the length of the right femur (Figure 1f). αActRIIA/IIB ab treatment led to a small, but significant, increase in femoral length (+2%) in ambulating mice, while this was not the case in BTX mice.

### 3.3 Smad2 Levels in Muscle Tissue

**FIGURE 2:**
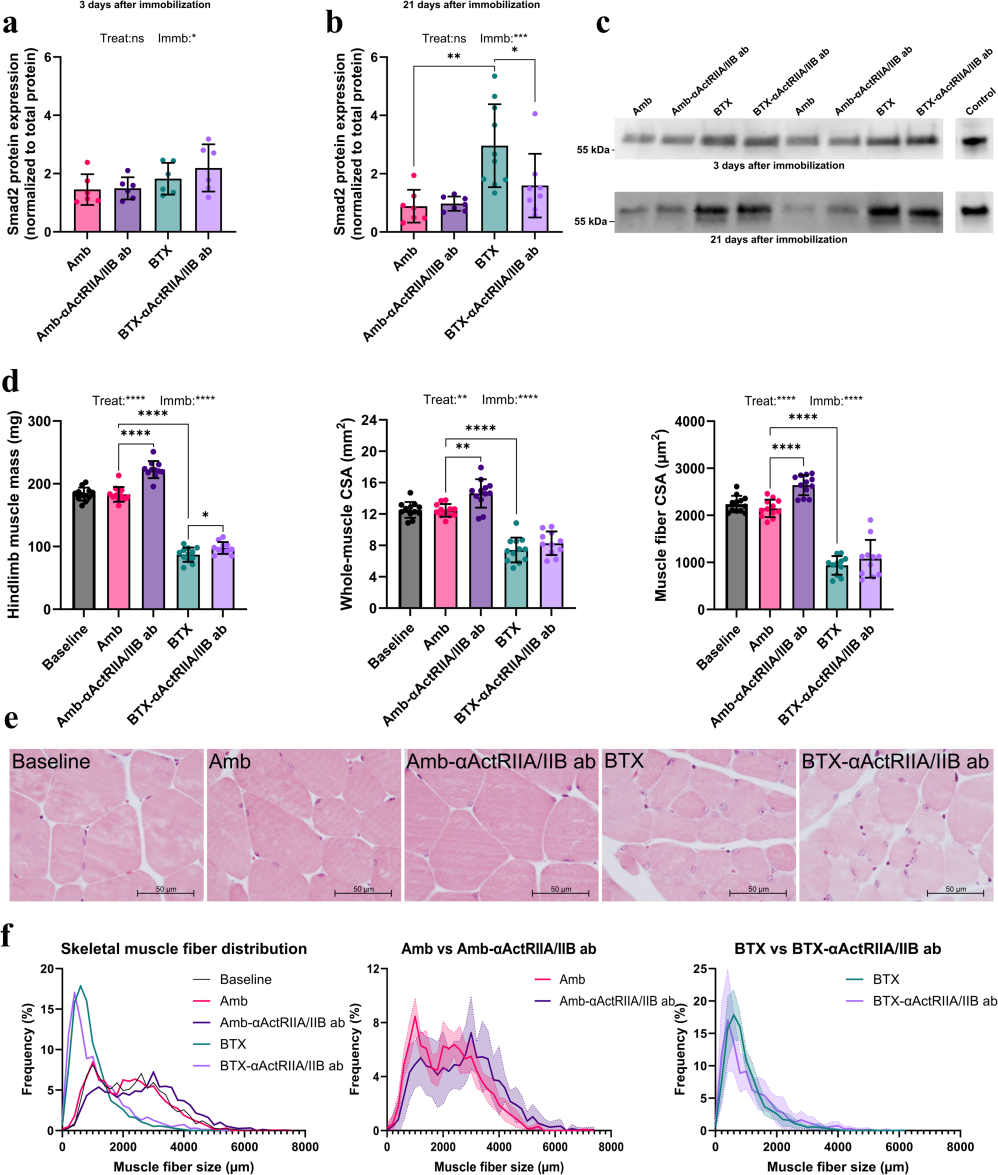
Muscle parameters. Relative Smad2 protein expression in gastrocnemius muscle tissue (a) 3 and (b) 21 days after immobilization. (c) Representative western blots of two animals from each group and positive control (from activin‐activated cell lysate) 3 and 21 days after immobilization. (d) Hindlimb muscle mass, whole‐muscle cross‐sectional area (CSA) of the rectus femoris muscle (RFM) and RFM muscle fibre CSA. Data is presented as mean ± SD. Key: Treat: effect of αActRIIA/IIB ab treatment in two‐way ANOVA. Immb: effect of BTX immobilization in two‐way ANOVA. *p < 0.05, **p < 0.01, ***p < 0.001, ****p < 0.0001, ns = not significant. (e) Masson trichrome stained cross‐sectional sections of the RFM. Each group is presented by the sample with muscle fibre CSA closest to the group average. (f) Frequency distribution of skeletal muscle fibre size of the RFM. Individual muscle fibre sizes have been clustered into bins of 200 μm. Presented is frequency distribution for all groups as well as for Amb versus Amb‐αActRIIA/IIB ab and for BTX versus αActRIIA/IIB ab. Solid lines show average cell size, and semitransparent areas show standard error.

There were no significant differences between groups in relative Smad2 protein expression in muscle tissue after 3 days (Figure 2a). However, after 21 days, BTX immobilization significantly increased Smad2 protein expression (+235%) in muscle tissue compared to ambulating animals (Figure 2b,c). Furthermore, treatment with αActRIIA/IIB ab for 21 days significantly decreased Smad2 protein expression (−46%) in immobilized mice but did not affect Smad2 levels in ambulating animals. We were unable to detect pSmad2 expression levels reliably in muscle tissue.

### 3.4 Muscle Mass and Muscle Fibre CSA

BTX reduced the combined mass of the right rectus femoris and gastrocnemius muscle (right hindlimb muscle mass) significantly compared to the Amb group (−53%) (Figure 2d). A similar reduction in rectus femoris whole muscle CSA (−40%) was found in BTX animals compared to Amb. The Amb‐αActRIIA/IIB ab group had a significantly increased right hindlimb muscle mass (+21%) as well as rectus femoris whole muscle CSA (+17%) compared to Amb mice. Similarly, αActRIIA/IIB ab treatment significantly increased right hindlimb muscle mass (+12%) for immobilized animals. However, rectus femoris whole muscle CSA did not differ between BTX‐αActRIIA/IIB ab and BTX.

Animals in the BTX group had a lower mean muscle fibre CSA (−54%) compared to those in the Amb group (Figure 2d,e). Conversely, Amb‐αActRIIA/IIB ab mice had significantly increased mean muscle fibre CSA (+23%) compared to Amb. No significant difference was found after αActRIIA/IIB ab treatment in the immobilized groups.

To investigate the effect of BTX and αActRIIA/IIB ab on skeletal muscle fibre CSA further, cell size frequency distributions were determined. BTX caused a clear shift in skeletal muscle fibre CSA towards smaller cellular sizes, whereas αActRIIA/IIB ab caused a shift towards larger cells, most pronounced in the ambulating mice (Figure 2f).

### 3.5 DEXA

**FIGURE 3:**
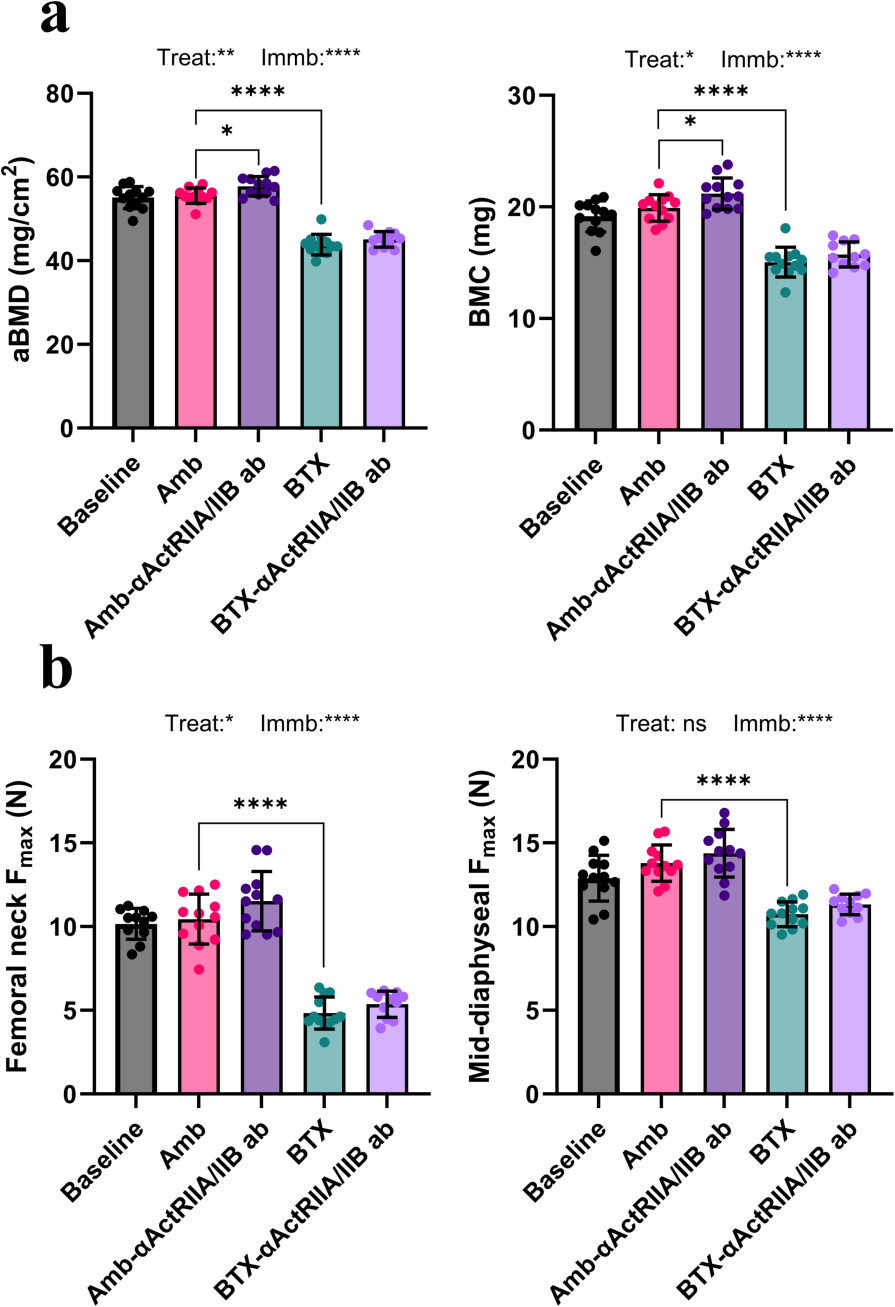
Femoral densitometry and strength. (a) Areal bone mineral density (aBMD) and bone mineral content (BMC) determined by dual‐energy x‐ray absorptiometry (DEXA) of the whole femur. (b) Bone fracture strength at the femoral neck and femoral mid‐diaphysis. Data is presented as mean ± SD. Key: Treat: effect of αActRIIA/IIB ab treatment in two‐way ANOVA. Immb: effect of BTX immobilization in two‐way ANOVA. *p < 0.05, **p < 0.01, ****p < 0.0001, ns = not significant.

BTX significantly decreased both aBMD (−21%) and BMC (−24%) of the whole femur compared to the Amb group (Figure 3a). Amb‐αActRIIA/IIB ab had a significantly increased femoral aBMD (+4%) and BMC (+6%) compared to Amb, but αActRIIA/IIB ab treatment could not counteract the effect of immobilization in BTX animals.

### 3.6 Mechanical Testing

BTX caused a significantly lower bone strength compared to Amb at both the femoral neck (−54%) and the femoral mid‐diaphysis (−22%) (Figure 3b). αActRIIA/IIB ab treatment did not lead to significantly different femoral neck strength in either the ambulating or immobilized mice separately. However, when combining the effect of αActRIIA/IIB ab treatment for both ambulating and immobilized animals in a two‐way ANOVA, there was a small but significant increase in femoral neck strength (*p* = 0.045).

### 3.7 μCT at the Femoral Distal Metaphysis

**FIGURE 4:**
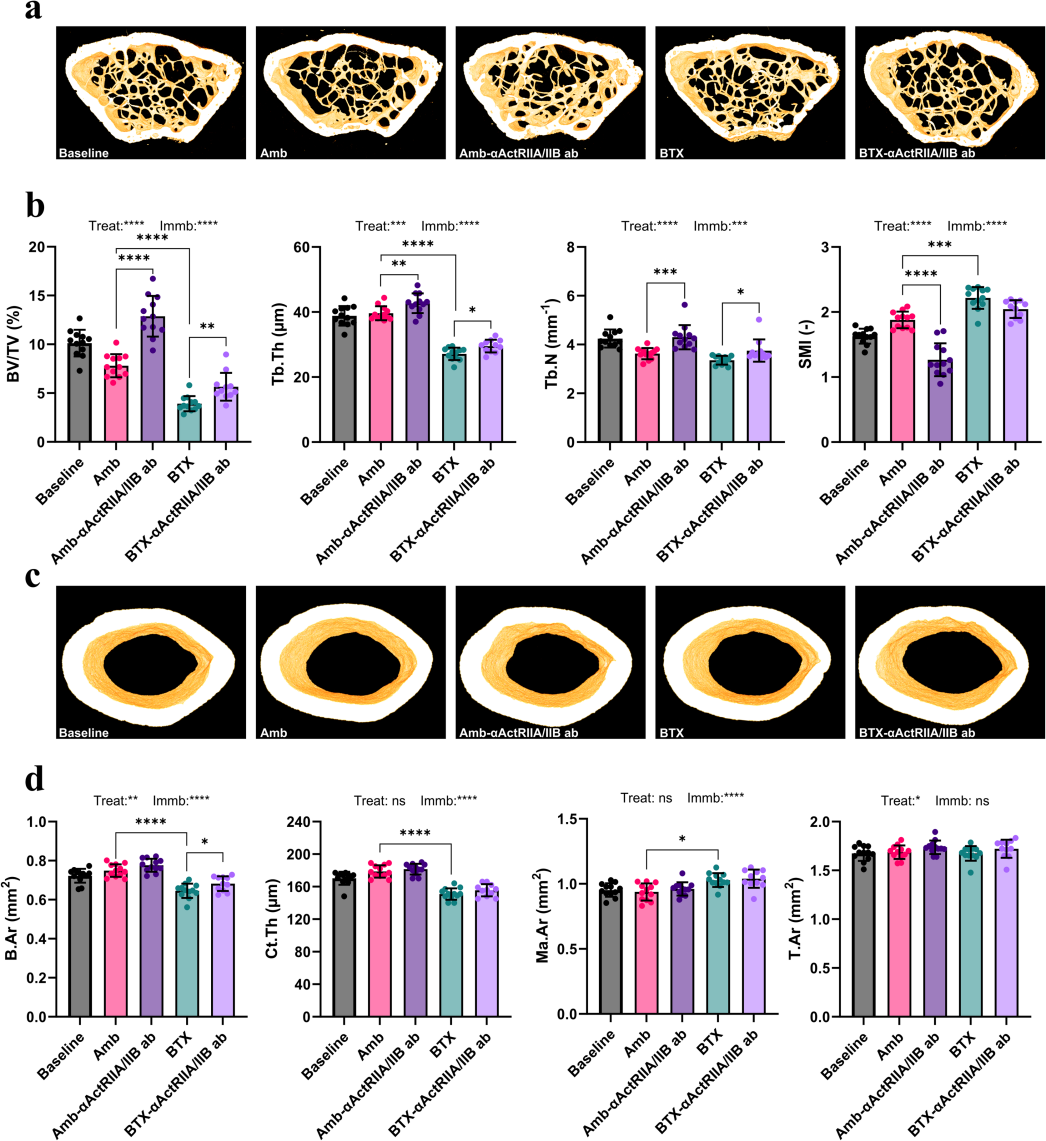
Bone μCT parameters. (a) 3D reconstructions of the distal femoral metaphysis. The samples presented from each group are those with bone volume fraction (BV/TV) closest to the group average. (b) BV/TV, trabecular thickness (Tb.Th), trabecular number (Tb.N) and structure model index (SMI) of the trabecular bone volume from the distal metaphysis. (d) 3D reconstructions of the femoral mid‐diaphysis. The samples presented from each group are those with bone area (B.Ar) closest to the group average. (e) Cortical B.Ar, cortical thickness (Ct.Th), marrow area (Ma.Ar) and tissue area (T.Ar) of the femoral mid‐diaphysis. Data is presented as mean ± SD. Key: Treat: effect of αActRIIA/IIB ab treatment in two‐way ANOVA. Immb: effect of BTX immobilization in two‐way ANOVA. *p < 0.05, **p < 0.01, ***p < 0.001, ****p < 0.0001, ns = not significant.

BTX caused a significant deterioration of the trabecular bone network at the distal femoral metaphysis compared to Amb. In particular, this manifested as a significant reduction of bone volume fraction (BV/TV: −50%) and trabecular thickness (Tb.Th: −32%) (Figure 4a,b). Furthermore, there was a significant increase in the structure model index (SMI: +18%), indicating that the shape of the trabeculae shifted from more plate‐like towards more rod‐like. Treatment with αActRIIA/IIB ab significantly increased BV/TV, Tb.Th and Tb.N in both ambulating (+65%, +8% and +19%, respectively) and immobilized (+44%, +9% and +12%, respectively) animals compared to their respective controls. SMI was significantly lower (−33%) for Amb‐αActRIIA/IIB ab compared to Amb, indicating a shift towards a more plate‐like trabecular structure.

### 3.8 μCT at the Femoral Mid‐Diaphysis

Animals injected with BTX had a significantly lower bone area (B.Ar: −14%) and cortical thickness (Ct.Th: −16%) compared to Amb (Figure 4c,d). Additionally, bone marrow area (Ma.Ar: +10%) was significantly increased in BTX compared to Amb. Treatment with αActRIIA/IIB ab significantly increased B.Ar in immobilized mice (+6%), while this did not reach the level of significance for ambulating mice (*p* = 0.064). αActRIIA/IIB ab treatment did not have any effect on either Ct.Th or M.Ar.

### 3.9 Osteoid, Osteoblasts, Osteoclasts and Adipocytes at Proximal Tibial Metaphysis

**FIGURE 5:**
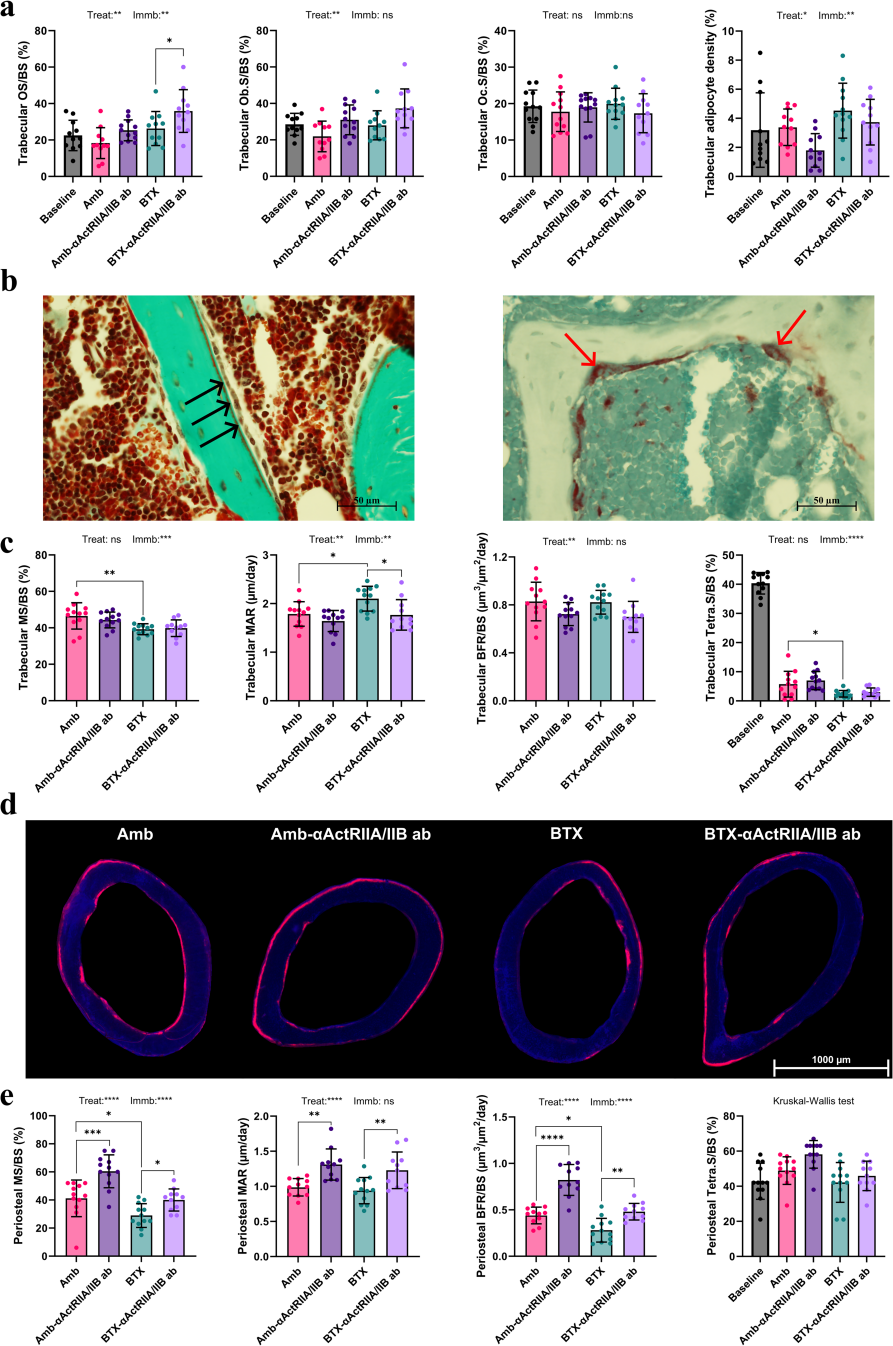
Bone histomorphometry. (a) Osteoid‐covered surfaces (OS/BS), osteoblast‐covered surfaces (Ob.S/BS), osteoclast‐covered surfaces (Oc.S/BS) and medullary adipocyte density of the trabecular bone at the proximal tibial metaphysis. (b) Images of a Masson–Goldner trichrome stained sample and a sample stained for TRAP, both at the proximal tibial metaphysis. Black arrows: osteoblasts. Red arrows: multinucleated osteoclasts. (c) Mineralizing surfaces (MS/BS), mineral apposition rate (MAR), bone formation rate (BFR/BS) and tetracycline‐covered surfaces (Tetra.S/BS) of the trabecular bone at the distal femoral metaphysis. (d) Dynamic histomorphometry at the femoral mid‐diaphysis. The blue areas are bone, and the pink areas show alizarin red incorporated into newly formed bone. The samples presented from each group are those with periosteal cortical MS/BS closest to the group average. (e) Mineralizing surfaces (MS/BS), mineral apposition rate (MAR), bone formation rate (BFR/BS) and tetracycline‐covered surfaces (Tetra.S/BS) of the femoral mid‐diaphyseal periosteal surface. Data is presented as mean ± SD. Key: Treat: effect of αActRIIA/IIB ab treatment in two‐way ANOVA. Immb: effect of BTX immobilization in two‐way ANOVA. *p < 0.05, **p < 0.01, ***p < 0.001, ****p < 0.0001, ns = not significant.

OS/BS did not differ between BTX and Amb (Figure 5a,b, Table S1). αActRIIA/IIB ab treatment caused a significant increase in OS/BS (+37%) after 21 days for immobilized mice, while no significant increase was found for ambulating mice. Ob.S/BS and Oc.S/BS did not differ between any of the groups.

BTX had no impact on medullary adipocyte density (Figure 5a, Table S1). While medullary adipocyte density did not decrease for animals treated with αActRIIA/IIB ab, there was nevertheless a tendency towards lower medullary adipocyte density for ambulating animals treated with αActRIIA/IIB ab (−47%, *p* = 0.054). A similar trend was seen for immobilized animals, although the effect was less pronounced (−17%, *p* = 0.220).

### 3.10 Femoral Dynamic Histomorphometry

At the distal femoral metaphyseal trabecular bone, BTX decreased MS/BS (−16%), increased MAR (+18%) and decreased Tetra.S/BS (−57%) compared to Amb (Figure 5c, Table S1). Contrary to expectation, treatment with αActRIIA/IIB ab significantly decreased MAR (−16%) for immobilized animals but had no effect on MS/BS, Tetra.S/BS or BFR/BS for either ambulating or immobilized mice.

At the femoral mid‐diaphyseal periostal bone, BTX significantly decreased MS/BS (−30%) and BFR/BS (−36%) compared to Amb (Figure 5d,e, Table S1). Animals treated with αActRIIA/IIB ab had significantly higher MS/BS (Amb‐αActRIIA/IIB ab vs. Amb: +46%, BTX‐αActRIIA/IIB ab vs. BTX: +39%), MAR (Amb‐αActRIIA/IIB ab vs. Amb: +33%, BTX‐αActRIIA/IIB ab vs. BTX: +31%) and BFR/BS (Amb‐αActRIIA/IIB ab vs. Amb: +87%, BTX‐αActRIIA/IIB ab vs. BTX: +71%) compared to their respective vehicle‐treated controls. Neither BTX nor αActRIIA/IIB ab affected Tetra.S/BS.

At the femoral mid‐diaphyseal endocortical bone, BTX significantly decreased MS/BS (−37%) (Table S1). Furthermore, BTX resulted in lower Tetra.S/BS (−41%) compared to Amb, indicating a higher amount of resorptive surfaces during the study period but did not affect MAR or BFR/BS. Treatment with αActRIIA/IIB ab had no effect on MS/BS, Tetra.S/BS, MAR or BFR/BS.

### 3.11 Haematological Parameters in 7‐Week‐Old and 19‐Week‐Old Mice

**FIGURE 6:**
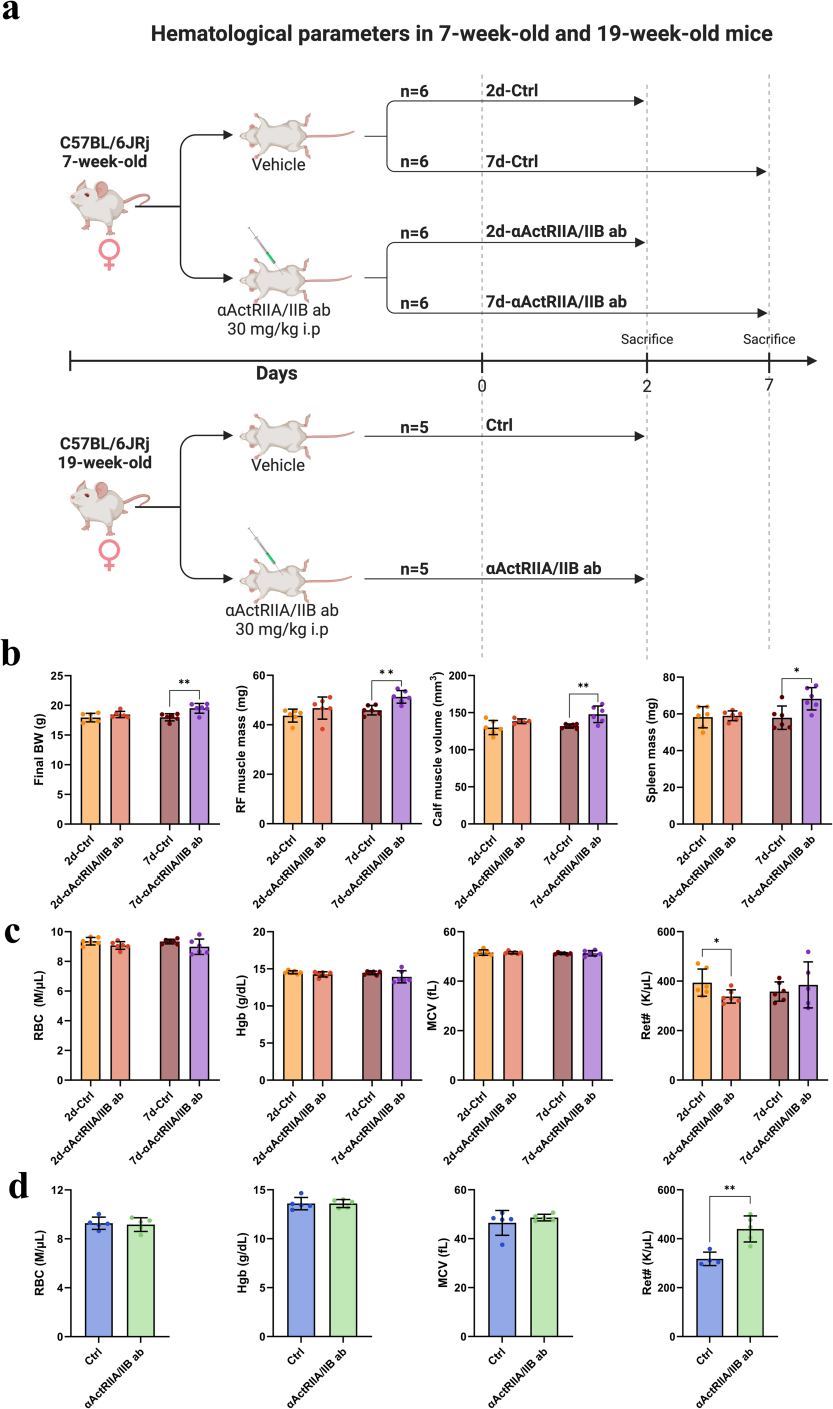
Haematological parameter substudies. (a) Study set‐up for the haematological substudies in 7‐week‐old and 19‐week‐old mice. Created with BioRender. (b) Final body weight (BW), left rectus femoris (RF) muscle mass, left calf muscle volume and spleen mass of 7‐week‐old mice 2 and 7 days after αActRIIA/IIB ab treatment. (c) Red blood cell count (RBC), haemoglobin (Hgb), mean cell volume (MCV) and reticulocyte count (Ret#) in 7‐week‐old mice 2 and 7 days after αActRIIA/IIB ab treatment. (d) RBC, Hgb, MCV and Ret# in 19‐week‐old mice 2 days after αActRIIA/IIB ab treatment. Data is presented as mean ± SD. *p < 0.05, **p < 0.01.

A visual representation of the experimental groups and study setup in both haematological substudies can be seen in Figure 6a.

To show that αActRIIA/IIB ab treatment was effective, BW and muscle mass were determined in the 7‐week‐old mice. Treatment with αActRIIA/IIB ab did not affect BW after 2 days. However, after 7 days, BW was significantly higher (+8%) for animals treated with αActRIIA/IIB ab than for Ctrls (Figure 6b).

Similarly, treatment with αActRIIA/IIB ab did not increase muscle mass or muscle volume after 2 days; however, 7 days after treatment, both rectus femoris muscle mass (+12%) and calf muscle volume (+12%) were significantly higher than Ctrls (Figure 6b).

Furthermore, the spleen mass was not affected 2 days after αActRIIA/IIB ab treatment for the 7‐week‐old mice, while the spleen mass was significantly higher (+18%) after 7 days for mice treated with αActRIIA/IIB ab than for Ctrls (Figure 6b). When the spleen mass was normalized for BW, there was still a tendency towards an increase in spleen mass 7 days after αActRIIA/IIB ab treatment (*p* = 0.081).

In 7‐week‐old mice, treatment with αActRIIA/IIB ab did not affect RBC, haemoglobin (HGB), haematocrit (HCT), mean cell volume (MCV), platelets or white blood cell count (WBC) after either 2 or 7 days (Figure 6c, Table 1). However, the 7‐week‐old mice had a significantly decreased reticulocyte count (−14%) 2 days, but not 7 days, after αActRIIA/IIB ab treatment.

**TABLE 1:** Haematological parameters. All haematological parameters of both 7‐week‐old and 19‐week‐old mice. Presented are red blood count (RBC), haemoglobin (HGB), haematocrit (HCT), mean cell volume (MCV), mean corpuscular haemoglobin (MCH), mean corpuscular haemoglobin concentration (MCHC), reticulocyte count (Ret#), platelets (PLT), white blood cell count (WBC), neutrophile leucocyte count (Neut#) and lymphocyte count (Lymph#).

	Haematological parameters in 7‐week‐old mice				Haematological parameters in 19‐week‐old mice	
	2d‐Ctrl	2d‐αAct‐RIIA/IIB ab	7d‐Ctrl	7d‐αAct‐RIIA/IIB ab	Ctrl	αAct‐RIIA/IIB ab
Animals (*n*)	6	6	6	6	5	5
RBC (M/μL)	9.4 (0.3)	9.1 (0.3)	9.3 (0.2)	9.0 (0.5)	9.3 (0.5)	9.2 (0.6)
HGB (g/dL)	14.5 (0.2)	14.3 (0.4)	14.5 (0.2)	13.9 (0.8)	13.6 (0.6)	13.6 (0.4)
HCT (%)	48.3 (1.6)	46.7 (1.4)	47.8 (0.7)	46.1 (2.1)	44.9 (2.1)	44.5 (1.6)
MCV (fL)	51.6 (1.1)	51.5 (0.5)	51.2 (0.3)	51.3 (1.1)	48.5 (1.2)	48.6 (1.3)
MCH (pg)	15.5 (0.2)	15.7 (0.2)	15.5 (0.1)	15.5 (0.2)	14.7 (0.2)	14.9 (0.6)
MCHC (g/dL)	30.1 (0.7)	30.5 (0.3)	30.3 (0.3)	30.2 (0.4)	30.3 (0.4)	30.6 (0.6)
RET# (K/μL)	394 (55)	338 (27)*	358 (39)	385 (93)	318 (28)	440 (53)**
PLT (K/μL)	391 (282)	561 (240)	845 (68)	824 (120)	817 (91)	636 (313)
WBC (K/μL)	6.35 (1.39)	5.27 (1.26)	5.91 (2.26)	5.51 (1.00)	7.94 (2.97)	7.14 (1.93)
Neut# (K/μL)	0.44 (0.12)	0.37 (0.10)	0.47 (0.24)	0.38 (0.08)	0.73 (0.34)	0.66 (0.34)
Lymph# (K/μL)	5.64 (1.20)	4.63 (1.04)	5.20 (1.96)	4.88 (0.86)	6.84 (2.50)	6.11 (1.52)

*Note:* Data is presented as mean (SD).

* *p* < 0.05 versus respective control.

** *p* < 0.01 versus respective control.

In 19‐week‐old mice, αActRIIA/IIB ab treatment did not affect the haematological parameters after 2 days (Figure 6d, Table 1). This was the case for RBC, HGB, HCT, MCV, platelet count and WBC. In contrast to the 7‐week‐old mice, the 19‐week‐old mice had a significantly increased reticulocyte count (+38%) 2 days after αActRIIA/IIB ab treatment.

## 4 Discussion

The present study showed that treatment with αActRIIA/IIB ab induced a substantial increase in muscle mass in ambulating mice, which is in agreement with previous studies in rodents and humans [17, 18, 19]. Furthermore, we showed that skeletal muscle hypertrophy was present as early as 7 days after αActRIIA/IIB ab administration in ambulating animals. We found that the muscle anabolic effect of αActRIIA/IIB ab treatment was less potent in immobilized animals than in ambulating animals. In part, this may be due to the catabolic impact of BTX‐induced muscle paresis, which may be so powerful that αActRIIA/IIB ab could not fully mitigate the response. This is supported by our finding that relative Smad2 protein expression increased during immobilization and decreased by αActRIIA/IIB ab treatment but did not seem to reach baseline. Similar findings were reported by Tando et al., who found that mechanical denervation‐induced immobilization increased Smad2 protein expression and that Smad2/3 knockout mice were resistant to immobilization‐induced skeletal muscle atrophy [32].

The study is, to our knowledge, the first to investigate the effects of αActRIIA/IIB ab on both bone and muscle parameters in osteopenic and sarcopenic mice as well as on haematopoiesis in mice.

In bone, αActRIIA/IIB ab induced an increase in aBMD, a strong bone anabolic response in trabecular bone, accompanied by a modest increase in bone formation in cortical bone. In addition, a two‐way ANOVA suggested that αActRIIA/IIB ab increased bone strength at the femoral neck, but the response was not potent enough to be detectable between separate groups in the multiple comparison analysis.

The small increase in cortical B.Ar stands in contrast to the large increase in MS/BS, MAR and BFR/BS at the cortical periosteal bone surfaces. However, this may be explained by the nature of the two methods of analysis. Where dynamic histomorphometry detects the adaptive bone response in a short time window around sacrifice, μCT shows the static change in total B.Ar, which has taken place during the entire treatment period. Thus, a longer treatment period may have further increased the cortical B.Ar. Furthermore, the lack of effect on the mid‐diaphyseal endocortical bone surface combined with a slightly increased tissue area and unchanged Ma.Ar suggests that on cortical bone, αActRIIA/IIB ab mainly acts on the periosteal surface.

The modest effect of αActRIIA/IIB ab on cortical bone found in the present study is in contrast to findings of similar studies of other IASPs, like activin ligand traps based on receptor IIA or IIB. Jeong et al. found that an Activin Receptor Type IIB ligand trap (10 mg/kg twice weekly) significantly increased cortical bone CSA for both wild‐type mice and mice suffering from osteogenesis imperfecta [33]. Similarly, we have shown that an Activin Receptor Type IIA ligand trap (10 mg/kg twice weekly) could significantly increase both B.Ar and Ct.Th in immobilization‐induced osteopenic mice [34]. The reason for the divergent effect on cortical bone between activin receptor ligand trap treatment and anti‐activin receptor antibodies is unknown. One explanation could be that αActRIIA/IIB ab also blocks the binding of bone morphogenetic proteins (BMPs) to the Activin Type 2 receptors and thus diverts BMP signalling to run mainly via Bone Morphogenetic Protein Receptor Type 2. This disturbance of the BMP signalling balance would not occur with activin receptor ligand traps, which is a major difference between the IASPs.

The effect of αActRIIA/IIB ab on bone fracture healing has been studied by Tankó et al., who found no effect of αActRIIA/IIB ab on callus formation or fracture healing [35]. Interestingly, they performed the bone fracture at the mid‐diaphysis, which comprises cortical bone only. Thus, the lack of effect of αActRIIA/IIB ab on mid‐diaphyseal bone healing is consistent with the findings of the present study that αActRIIA/IIB ab mainly exerts its anabolic effect on trabecular bone, while the effect on cortical bone is limited.

The bioactivity and receptor‐blocking ability of αActRIIA/IIB ab were quantified in the present study using a cell‐based pSmad2/3 assay. The findings aligned with previous studies evaluating the activin receptor pathway [36, 37] and were further supported by Lach‐Trifilieff et al., who found that myostatin stimulation of human myoblasts in vitro increased pSmad2 and pSmad3 protein expression and that the addition of αActRIIA/IIB ab inhibited this [16]. Our study sought to extend these findings with in vivo evidence on the effect of αActRIIA/IIB ab on Smad pathway regulation. Canonical activin signalling involves Smad protein phosphorylation. However, our in vivo analysis encountered a limitation: we could only measure combined Smad2 (phosphorylated and nonphosphorylated) reliably in muscle tissue, whereas pSmad2 levels were challenging to detect. Although pSmad2 detection via western blotting is well‐established in cell studies, there appear to be few in vivo studies that successfully detect pSmad2 in the lysate of skeletal muscle tissue [32, 38]. To our knowledge, no studies have yet demonstrated detectable pSmad2 in muscle tissue following αActRIIA/IIB ab treatment.

The impact of αActRIIA/IIB ab treatment on haematological parameters was very sparse. There was no effect on RBC, neither for 7‐week‐old mice nor for 19‐week‐old mice. However, the spleen mass was increased 7 days after αActRIIA/IIB ab treatment. This increase may be due to either a direct effect of αActRIIA/IIB ab on the spleen or a secondary effect caused by a general increase in BW or muscle mass, for example, due to increased blood volume.

These findings on haematopoiesis for αActRIIA/IIB ab differ from those found for activin receptor ligand trap treatment, where increased haematopoiesis has been found in both mice and humans [14, 39]. In fact, Carrancio et al. showed that treatment with an activin receptor IIA ligand trap (30 mg/kg) caused a significant increase in RBC, Hb and HCT both after 2 and 7 days in 6–8‐week‐old female C57 mice [14]. Furthermore, they found an increased reticulocyte count after 7 days. Studies investigating the effect of the activin receptor IIB ligand trap instead of IIA found similar results [40]. The disparate effect on haematopoiesis may be due to the different methods of action, where αActRIIA/IIB ab targets and blocks the activin receptor directly and the ligand traps instead bind the soluble ligands.

In conclusion, treatment with αActRIIA/IIB ab rapidly and potently increased muscle mass in ambulating animals and alleviated some of the muscle loss caused by immobilization. Moreover, αActRIIA/IIB ab demonstrated a potent anabolic response on trabecular bone and a more modest response on cortical bone. Finally, short‐term treatment with αActRIIA/IIB ab had no effect on haematopoiesis but increased spleen mass.

## Ethics Statement

The authors have nothing to report.

## Conflicts of Interest

The authors declare no conflicts of interest.

## Supporting information


**Table S1** Histological properties of the bone. Histological properties were obtained at the femoral mid‐diaphysis, the distal femoral metaphysis and the proximal tibial metaphysis. Presented is bone mineralizing surface (MS/BS), mineral apposition rate (MAR), bone formation rate (BFR/BS), tetracycline‐covered bone surfaces (Tetra.S/BS), osteoid‐covered bone surfaces (OS/BS), osteoblast‐covered bone surfaces (Ob.S/BS), osteoclast‐covered surfaces (Oc.S/BS) and medullary adipocyte density (N.Ad/N.Ma). Data is presented as mean (SD). ^a^ *p* < 0.05 for Amb versus BTX. ^b^ *p* < 0.05 for Amb versus Amb‐αActRIIA/IIB ab. ^c^ *p* < 0.05 for BTX versus BTX‐αActRIIA/IIB ab.

**Data S1** Supporting information.
