# Supplementary material for: The Effect of Anti‐Activin Receptor Type IIA and Type IIB Antibody on Muscle, Bone and Blood in Healthy and Osteosarcopenic Mice
Source: J Cachexia Sarcopenia Muscle. 2025 Jan 30;16(1):e13718. doi: 10.1002/jcsm.13718 (PMC11780395; doi:10.1002/jcsm.13718)
Supplement: Supplementary file 1 — Table S1 Histological properties of the bone. Histological properties were obtained at the femoral mid‐diaphysis, the distal femoral metaphysis and the proximal tibial metaphysis. Presented is bone mineralizing surface (MS/BS), mineral apposition rate (MAR), bone formation rate (BFR/BS), tetracycline‐covered bone surfaces (Tetra.S/BS), osteoid‐covered bone surfaces (OS/BS), osteoblast‐covered bone surfaces (Ob.S/BS), osteoclast‐covered surfaces (Oc.S/BS) and medullary adipocyte density (N.Ad/N.Ma). Data is presented as mean (SD). a p < 0.05 for Amb versus BTX. b p < 0.05 for Amb versus Amb‐αActRIIA/IIB ab. c p < 0.05 for BTX versus BTX‐αActRIIA/IIB ab. [file JCSM-16-e13718-s001.docx]

**Supplementary table 1 Histological properties of the bone** Histological properties obtained at the femoral mid-diaphysis, the distal femoral metaphysis, and the proximal tibial metaphysis. Presented is bone mineralizing surface (MS/BS), mineral apposition rate (MAR), bone formation rate (BFR/BS), tetracycline-covered bone surfaces (Tetra.S/BS), osteoid-covered bone surfaces (OS/BS), osteoblast-covered bone surfaces (Ob.S/BS), osteoclast-covered surfaces (Oc.S/BS), and medullary adipocyte density (N.Ad/N.Ma). Data is presented as mean (SD). a *p* < 0.05 for Amb vs. BTX. b *p* < 0.05 for Amb vs. Amb-αActRIIA/IIB ab. c *p* < 0.05 for BTX vs. BTX-αActRIIA/IIB ab.

|  | **Baseline** | **Amb** | **Amb-αAct-**  **RIIA/IIB ab** | **BTX** | **BTX-** **αAct-**  **RIIA/IIB ab** |  |
| --- | --- | --- | --- | --- | --- | --- |
| ***Periosteal cortex at the femoral mid-diaphysis*** | | | | | | |
| **MS/BS (%)** | **-** | **41.3 (13.0)** | **60.4 (11.5)^b^** | **28.8 (8.4)^a^** | **40.0 (7.7)^c^** |  |
| **MAR (µm/d)** | **-** | **0.99 (0.12)** | **1.31 (0.22)^b^** | **0.94 (0.19)** | **1.23 (0.26)^c^** |  |
| **BFR/BS (µm^3^/µm^2^/d)** | **-** | **0.44 (0.09)** | **0.82 (0.17)^b^** | **0.28 (0.13)^a^** | **0.48 (0.09)^c^** |  |
| **Tetra.S/BS (%)** | **42.7 (10.4)** | **48.9 (7.9)** | **58.0 (7.8)** | **42.0 (11.4)** | **45.8 (8.5)** |  |
|  |  |  |  |  |  |  |
| ***Endosteal cortex at the femoral mid-diaphysis*** | | | | | | |
| **MS/BS (%)** | **-** | **46.3 (15.3)** | **49.5 (12.3)** | **29.1 (10.3)^a^** | **36.8 (8.3)** |  |
| **MAR (µm/d)** | **-** | **1.04 (0.23)** | **0.94 (0.13)** | **1.39 (0.31)** | **1.21 (0.24)** |  |
| **BFR/BS (µm^3^/µm^2^/d)** | **-** | **0.49 (0.21)** | **0.47 (0.17)** | **0.41 (0.09)** | **0.45 (0.15)** |  |
| **Tetra.S/BS (%)** | **59.7 (17.3)** | **45.8 (10.5)** | **48.6 (7.6)** | **26.9 (14.1)^a^** | **30.1 (7.3)** |  |
|  |  |  |  |  |  |  |
| ***Trabecular properties at the femoral distal metaphysis*** | | | | | | |
| **MS/BS (%)** | **-** | **46.5 (7.3)** | **44.3 (4.3)** | **39.3 (3.0)^a^** | **39.8 (4.6)** |  |
| **MAR (µm/d)** | **-** | **1.79 (0.25)** | **1.64 (0.22)** | **2.1 (0.25)^a^** | **1.77 (0.31)^c^** |  |
| **BFR/BS (µm^3^/µm^2^/d)** | **-** | **0.83 (0.16)** | **0.72 (0.10)** | **0.82 (0.10)** | **0.70 (0.13)** |  |
| **Tetra.S/BS (%)** | **40.3 (3.7)** | **5.7 (4.4)** | **7.0 (3.1)** | **2.4 (1.1)^a^** | **3.0 (1.4)** |  |
|  |  |  |  |  |  |  |
| ***Trabecular properties at the tibial proximal metaphysis*** | | | | | |  |
| **OS/BS (%)** | **22.5 (8.3)** | **20.3 (10.6)** | **25.3 (5.7)** | **26.3 (9.3)** | **35.9 (11.7)^c^** |  |
| **Ob.S/BS (%)** | **28.6 (6.0)** | **24.1 (11.2)** | **31.0 (8.2)** | **28.0 (8.0)** | **37.3 (10.7)** |  |
| **Oc.S/BS (%)** | **19.3 (4.4)** | **17.8 (5.4)** | **19.0 (4.0)** | **20.0 (4.3)** | **17.4 (5.3)** |  |
| **N.Ad/N.Ma (%)** | **3.2 (2.6)** | **4.2 (3.2)** | **2.1 (1.5)** | **4.5 (1.9)** | **3.7 (1.6)** |  |
