# Supplementary material for: The Effect of Anti‐Activin Receptor Type IIA and Type IIB Antibody on Muscle, Bone and Blood in Healthy and Osteosarcopenic Mice
Source: J Cachexia Sarcopenia Muscle. 2025 Jan 30;16(1):e13718. doi: 10.1002/jcsm.13718 (PMC11780395; doi:10.1002/jcsm.13718)
Supplement: Supplementary file 2 — Data S1 Supporting information. [file JCSM-16-e13718-s002.docx]

**3.1 Preparation of anti-activin receptor type IIA/IIB antibody**

Two constructs, one encoding the light chain and the other encoding the IgG1 heavy chain of αActRIIA/IIB ab, were synthesized and cloned (GenScript Biotech, Rijswijk, Netherlands) into the vectors pIRES2-ZsGreen (#632478, Takara Bio, Saint-Germain-en-Laye, France) and pIRES2-DsRed-Express2 (#632540, Takara Bio, Saint-Germain-en-Laye, France), respectively. In both vectors, the constructs were preceded by a fragment encoding the serum albumin preproprotein signal peptide. The vectors were co-transfected into Chinese hamster ovary (CHO) cells (#R80007, FreeStyle CHO-S, Gibco, Thermo Fisher Scientific, MA, USA) and a stable cell line was established. The αActRIIA/IIB ab was purified from the culture medium using protein A affinity chromatography according to the manufacturer’s protocol (#17040301, HiTrap Protein A, Cytiva Europe, Germany). αActRIIA/IIB ab was exchanged into PBS and purity was estimated to be > 95% by SDS-PAGE. Bioactivity was evaluated in a reporter assay of HEK293 cells containing a (CAGA)12-luciferase sequence as a readout for phorsphorylated (p)Smad2/3 signaling, as previously described [1].

**3.3 Western blot analysis**

To investigate the *in vivo* effect of both immobilization and αActRIIA/IIB ab on the activin signaling pathway, western blot analysis was performed to assess relative Smad2 protein expression. The gastrocnemius muscles were homogenized in a lysis buffer of radio-immunoprecipitation assay (RIPA) buffer (#89901, RIPA lysis and extraction buffer, Thermo Fisher Scientific, MA, USA) containing protease inhibitor (#11836153001, cOmplete, Mini Protease Inhibitor Cocktail, Sigma-Aldrich, St. Louis, MO, USA) and phosphatase inhibitor (#4906845001, PhosSTOP, Sigma-Aldrich, St. Louis, MO, USA). The homogenized samples were agitated for 2 hours at 4°C and centrifuged at 16.000 × g for 20 mins. The supernatant was collected, and protein concentration was determined using a Bradford assay (#23200, Pierce Bradford Protein Assay Kit, Thermo Fisher Scientific, MA, USA). For Smad2, 30 µg of protein from each animal was loaded onto a gel for SDS-PAGE separation and stain-free visualization on an imaging system (Gel Doc EZ Imager; Bio-Rad Laboratories, Hercules, California, USA). Subsequently, proteins were transferred to a PVDF membrane (#IB401032, iBlot Transfer Stack, PVDF, mini, Thermo Fisher Scientific, MA, USA), blocked for 1 hour in tris-buffered saline with 0.1% Tween (TBST) with 5% w/v nonfat dry milk, incubated overnight with primary antibody (anti-Smad2, #5339, Smad2 (D43B4) XP Rabbit mAb; Cell Signaling Technology Inc., Danvers, MA, USA and anti-pSmad2, #3108, Phospho-Smad2 (Ser465/467) (138D4) Rabbit mAb; Cell Signaling Technology Inc., Danvers, MA, USA) in TBST with 5% w/v bovine serum albumin, and then incubated for 1 hour with secondary antibody (#A0545, Anti-Rabbit IgG, Sigma-Aldrich, St. Louis, MO, USA) in TBST with 5% w/v nonfat dry milk. The blots were digitally developed using an imaging system (ImageQuant LAS 4000; GE Healthcare, Chicago, Illinois, USA). Bands for Smad2 were quantified using FIJI and normalized to the total protein load, as determined from the stain-free gel analysis [2].

**3.8 Micro-computed tomography of bone**

Cortical and trabecular bone structure of the right femora were determined using micro-computed tomography (µCT) (µCT35; Scanco Medical AG, Brüttisellen, Switzerland) using an X-ray tube voltage of 55 kV and current of 145 µA. Both the distal metaphysis and the mid-diaphysis were imaged, and a volume of interest was selected for each skeletal site, as previously described [3]. The distal metaphysis and the mid-diaphysis were imaged in high-resolution mode (1000 projections/180°) using a voxel-size of 3.5 µm and 7 µm and an integration time of 800 ms and 300 ms, respectively. Data was segmented using a threshold of 531 mg hydroxyapatite (HA)/cm^3^ and 573.7 mg HA/cm^3^ for the femoral distal metaphysis and mid-diaphysis, respectively.

**3.9 Bone preparation, cell counting, and dynamic histomorphometry**

After mechanical testing, a 200-µm-thick cross section was cut from the mid-diaphysis of the right femur, mounted on a glass slide, and left unstained. Thereafter, the remaining right femur was placed in 4% formaldehyde for 48 h and then transferred to 70% ethanol.

Subsequently, the right distal femur and the right tibia were embedded undecalcified in methylmetacrylate, cut into 7-µm-thick longitudinal sections, and mounted on glass slides. The femoral samples were left unstained, while the tibial samples were stained with either Masson-Goldner trichrome or enzymatically stained for tartrate-resistant acid phosphatase (TRAP).

All histological samples were assessed by a group-blinded investigator using a light microscope equipped with fluorescent light (Eclipse i80; Nikon, Tokyo, Japan) connected to a computer with Visiopharm stereology software (Visiopharm v. 2020.09.0.8195; Visiopharm, Hoersholm, Denmark). A total magnification of ×1027 was used.

At the proximal tibial metaphysis, osteoid-covered bone surfaces (OS/BS), osteoblast-covered bone surfaces (Ob.S/BS), and medullary adipocyte density were estimated on Masson-Goldner trichrome stained sections, while osteoclast-covered bone surfaces (Oc.S/BS) were estimated on sections stained for TRAP, as previously described [4].

Mineralizing surface (MS/BS), mineral apposition rate (MAR), and bone formation rate (BFR/BS) were estimated at the femoral mid-diaphysis and metaphysis [5]. In short, MS/BS is the fraction of bone surfaces covered with fluorescent labels, indicating surfaces with active bone formation. MAR is the distance between alizarin double labels divided by the time between labels (4 days), indicating the amount of daily bone formation at specific sites. BFR/BS is determined as MS/BS × MAR and indicates the daily amount of bone added at all bone surfaces. Furthermore, tetracycline labelled bone surfaces (Tetra.S/BS) were counted to estimate bone surface erosion over the entire study duration.

**References**

1. Meier D, Lodberg A, Gvozdenovic A, Pellegrini G, Neklyudova O, Born W *et al.* Inhibition of the activin receptor signaling pathway: A novel intervention against osteosarcoma. *Cancer Med* 2021;**10**:286–296.

2. Maloy A, Alexander S, Andreas A, Nyunoya T, Chandra D. Stain-Free total-protein normalization enhances the reproducibility of Western blot data. *Anal Biochem* 2022;**654**:114840.

3. Bromer FD, Brent MB, Pedersen M, Thomsen JS, Brüel A, Foldager CB. The Effect of Normobaric Intermittent Hypoxia Therapy on Bone in Normal and Disuse Osteopenic Mice. *High Alt Med Biol* 2021;**22**:225–234.

4. Brent MB, Thomsen JS, Brüel A. Short-term glucocorticoid excess blunts abaloparatide-induced increase in femoral bone mass and strength in mice. *Sci Rep* 2021;**11**:1–15.

5. Dempster DW, Compston JE, Drezner MK, Glorieux FH, Kanis JA, Malluche H *et al.* Standardized nomenclature, symbols, and units for bone histomorphometry: A 2012 update of the report of the ASBMR Histomorphometry Nomenclature Committee. *J Bone Miner Res* 2013;**28**:2–17.
